# Lipid nanoemulsions and liposomes improve photodynamic treatment efficacy and tolerance in CAL-33 tumor bearing nude mice

**DOI:** 10.1186/s12951-016-0223-8

**Published:** 2016-10-03

**Authors:** Doris Hinger, Susanna Gräfe, Fabrice Navarro, Bernhard Spingler, Devaraj Pandiarajan, Heinrich Walt, Anne-Claude Couffin, Caroline Maake

**Affiliations:** 1 Institute of Anatomy, University of Zurich, Winterthurerstrasse 190, Zurich, 8057 Switzerland; 2 Biolitec Research GmbH, Otto-Schott-Str. 15, 07745 Jena, Germany; 3 CEA, LETI, MINATEC Campus, Commissariat à l’Énergie Atomique et aux Énergies Alternatives (CEA), Technologies for Biology and Healthcare Division, 38054 Grenoble, France; 4 Université Grenoble Alpes, Grenoble, 38000 France; 5 Department of Inorganic Chemistry, University of Zurich, Winterthurerstrasse 190, Zurich, 8057 Switzerland; 6 Department of Cranio-Maxillofacial Surgery, University Hospital Zurich, Frauenklinikstrasse 24, Zurich, 8091 Switzerland

**Keywords:** Nanoemulsion, mTHPC, Head and neck cancer, PDT, Liposome

## Abstract

**Background:**

Photodynamic therapy (PDT) as promising alternative to conventional cancer treatments works by irradiation of a photosensitizer (PS) with light, which creates reactive oxygen species and singlet oxygen (^1^O_2_), that damage the tumor. However, a routine use is hindered by the PS’s poor water solubility and extended cutaneous photosensitivity of patients after treatment. In our study we sought to overcome these limitations by encapsulation of the PS m-tetrahydroxyphenylchlorin (mTHPC) into a biocompatible nanoemulsion (Lipidots).

**Results:**

In CAL-33 tumor bearing nude mice we compared the Lipidots to the existing liposomal mTHPC nanoformulation Foslip and the approved mTHPC formulation Foscan. We established biodistribution profiles via fluorescence measurements in vivo and high performance liquid chromatography (HPLC) analysis. All formulations accumulated in the tumors and we could determine the optimum treatment time point for each substance (8 h for mTHPC, 24 h for Foslip and 72 h for the Lipidots). We used two different light doses (10  and 20 J/cm^2^) and evaluated immediate PDT effects 48 h after treatment and long term effects 14 days later. We also analyzed tumors by histological analysis and performing reverse transcription real-time PCR with RNA extracts. Concerning tumor destruction Foslip was superior to Lipidots and Foscan while with regard to tolerance and side effects Lipidots were giving the best results.

**Conclusions:**

We could demonstrate in our study that nanoformulations are superior to the free PS mTHPC. The development of a potent nanoformulation is of major importance because the free PS is related to several issues such as poor bioavailability, solubility and increased photosensibility of patients. We could show in this study that Foslip is very potent in destroying the tumors itself. However, because the Lipidots' biocompatibility is outstanding and superior to the liposomes we plan to carry out further investigations and protocol optimization. Both nanoformulations show great potential to revolutionize PDT in the future.

**Electronic supplementary material:**

The online version of this article (doi:10.1186/s12951-016-0223-8) contains supplementary material, which is available to authorized users.

## Background

Photodynamic therapy (PDT) has received more attention in recent years as attractive alternative to conventional cancer treatments such as chemotherapy, surgery or radiotherapy [1, 2]. The principle of photodestruction on which it relies on works by exposure of a so called photosensitizer (PS) to light of an appropriate wavelength, which in turn depends on the kind of PS used. The PS reacts with oxygen upon irradiation and generates reactive oxygen species (ROS) and singlet oxygen (^1^O_2_) which damage surrounding tissue [3–5]. However, the short lifetime of singlet oxygen (<0.04 µs) and low diffusion potential with a small radius of action (0.02 µm) limits the damage to the irradiated spot [6]. In addition to this direct killing of malignant cells [7] tumor destruction can also be accomplished by targeting the tumor associated vessels [8]. However, a third possibility is to create longer lasting effects via stimulation of the immune system which subsequently may prevent tumor recurrence [9].

PDT is a strictly local modality that offers certain advantages over established anti-cancer regimes. It is e.g. minimally invasive, does not have a maximal lifetime dose and can therefore be repeated [1], gives an excellent cosmetic and functional outcome [10], does not produce drug resistance [11], and is not associated with severe systemic side effects [12]. PSs are preferentially taken up by malignant cells and therefore exhibit an inherent selectivity [13]. For this reason PSs can also be used as imaging probes in photodiagnosis (PDD) [14]. However, the selectivity leaves still room for improvement. The currently most widely used PSs are porphyrin derivatives. In an attempt to improve their optical properties modifications to the porphyrin structure have been made and led to the discovery of several second generation PSs like phthalocyanines [15] and chlorins [16]. The powerful chlorin PS m-tetrahydroxyphenylchlorin (mTHPC) is a well characterized substance and was highly successful in various in vitro, in vivo studies and clinical trials which has ultimately led to its approval for palliative treatment of head and neck cancer in Europe [17–20]. Although very promising its routine use in the clinic is hampered by poor water solubility which leads to aggregation, problematic systemic administration and suboptimal biodistribution. Moreover extended photosensitivity of patients after treatment impairs applicability [5, 21].

A possible solution to these drawbacks can be offered by encapsulation of PSs into nanocarriers. With this approach several problems could be tackled at once. First of all the solubility can be drastically improved, easing intravenous injections. Furthermore cancer selectivity could be increased by passive targeting, profiting from the enhanced permeability and retention effect (EPR) of nanoparticles in solid tumors [22]. Due to the high payload of nanoformulations, accumulation of greater pharmacological PS doses within the tumor may be facilitated, which could improve PDT effects by lowering the risk of an unwanted photosensitivity of healthy tissues, such as the skin.

We recently developed a biocompatible nanoemulsion (Lipidots) [23] as carrier for mTHPC with excellent optical properties which we subsequently tested in two in vitro studies [24, 25]. It could be demonstrated that Lipidots can significantly lower the dark toxicity of mTHPC while maintaining its photodynamic activity. In the course of our research we identified the most promising Lipidot formulation which we decided to further test in vivo.

In the present study we compared this novel mTHPC nanoemulsion (Lipidots) with a liposomal mTHPC formulation (Foslip) [26], which has been shown to produce promising results with regard to tumor destruction in cats [27, 28], and the approved mTHPC formulation (Foscan), in CAL-33 tumor bearing nude mice.

## Methods

### Drug and nanoparticle preparation

MTHPC and the liposomal mTHPC formulation Foslip were obtained from Biolitec Research GmbH, Jena, Germany as powder. A stock solution of 1 mg/mL Foscan was prepared by dissolving the mTHPC powder in a 40/60 ethanol/propylene glycol mixture and filtered through a syringe filter (0.22 µm pore size; TPP, Trasadingen, Switzerland). Foslip (20 mg/mL DPPC/DPPG, 2.2 mM mTHPC, 50 mg/mL Glucose) was reconstituted with sterile water, giving a stock solution of 1.5 mg/mL (≙2.2 mM) mTHPC content, with an average particle size of 135 nm and a polydispersity index (PDI) of 0.089. A nanoemulsion containing mTHPC (Lipidots, 50 mg/mL lipid, 1.06 mM mTHPC) with an average particle diameter of 50 nm and a PDI of 0.17 was prepared according to Delmas et al. [23] and Navarro et al. [24].

Briefly, Lipidots were manufactured by selecting the suitable weight ratios of core/shell excipients to design 50 nm diameter nanoparticles. The dispersion is composed of 37.5 % (w/w) of lipid phase (with a lecithin/PEG surfactant weight ratio of 0.19 and a surfactant/core weight ratio of 1.20). The Lipidots were loaded with 920 molecules of mTHPC/particle. MTHPC was incorporated into the lipid mixture as a concentrated solution in ethyl acetate and after vacuum elimination of organic solvent, the oily phase was added to the aqueous phase and emulsification was performed as previously described [24]. The mTHPC concentrations were determined by high-performance liquid chromatography (HPLC) analysis. Separation was achieved on a Sunfire C18 column (250 mm × 4.6 mm, i.d. 5 µm) at 30 °C. The mTHPC compound was eluted at 2.10 min using a isocratic mobile phase of acetonitrile/H_2_O trifluoroacetic acid, 0.1 %: 9/1 at 1 mL/min flow rate after injection of 30 µL. The UV detection is operated at 425 nm. The mTHPC concentrations were assessed using a calibration curve in the range of 1–12 µg/mL. Physicochemical characterization data of Lipidots can be found in the supplements (Additional file 1: Table S1).

All solutions were stored at four degrees Celsius in the dark and further diluted with sterile phosphate buffered saline (PBS) for injection (0.15 mg/kg mTHPC).

If not otherwise indicated, all chemicals were purchased from Sigma-Aldrich, Buchs, Switzerland.

### Cell culture

CAL-33, tongue squamous cell carcinoma cells (DSMZ, Braunschweig, Germany), were grown in RPMI-1640 medium without phenol red and with 10 % fetal calf serum (FCS), 2 mM Glutamax (Life Technologies, Carlsbad, USA), 1 % Penicillin/Streptomycin as supplements. Cells were kept in 75 cm^2^ cell culture flasks at 5 % CO_2_ and 37 °C. Cell counting was performed with a Neubauer chamber (Laboroptik Ltd., Lancing, UK) on an aliquot of cells after staining with 0.1 % (w/v) nigrosin in PBS.

### Husbandry conditions of mice & tumor model

Female immune deficient CD1-*Foxn1*^*nu*^ nude mice (4-6 weeks old) were obtained from Charles River, Sulzfeld, Germany. The mice were kept as groups of 5 in individually ventilated cages (IVC) under specific pathogen free (SPF) conditions and provided with food and water ad libitum.To establish the tumor model 9 mice each were subcutaneously injected into the right flank with 1.0 × 10^6^, 1.5 × 10^6^ or 2.0 × 10^6^ CAL-33 cells in 0.1 mL ringer lactate (Kantonsapotheke, Zurich, Switzerland) using a 26 G needle and one mL syringe (B. Braun, Melsungen, Germany). The animals were examined at least every third day for up to 42 days. Upon examination the mice were weighed and scored for abnormalities in behavior and appearance. Tumor sizes were measured with a Vernier caliper.

All animal experiments were implemented with approval of the Swiss cantonal ethics committee for animal experiments (No. 156/2012).

### Biodistribution studies

To determine pharmacokinetics Foscan, Foslip and Lipidots were injected intravenously into 10 mice each at a final concentration of 0.15 mg mTHPC/kg bodyweight (bw). Fluorescence measurements were carried out four, 8, 12, 24, 48 and 72 h after drug injection, by pressing the optic fiber of a spectrometer (PDT fluorometer; JETI Technische Instrumente GmbH, Jena, Germany) on three different spots on the tumor while holding the mice restrained. Three different spots on the skin were also analyzed as a reference. After the last measurement the mice were sacrificed and the tissues (tumor, skin, liver, spleen, kidney) were weighed, cut in small pieces and snap frozen in liquid nitrogen. For HPLC analysis the tissue was freeze dried (Christ Freeze drying system Alpha 1–4 LSC). The resulting powdered tissue was weighed and approximately 10–20 mg was transferred to a two milliliter reaction tube. Then 1.5 mL of methanol:DMSO (3:5, v:v) was added followed by immediate mixing for three times five sec using a vortex mixer (Merck Eurolab, MELB 1719) operating at 2400 rpm and then incubated at 60 °C while continuously shaking for at least 12 h. All samples were then spun at 16,000*g* in a centrifuge (Microfuge, Heraeus, Germany) for 5 min. One milliliter of each supernatant was transferred to a HPLC vial and analysed by HPLC.The HPLC system consisted of the solvent module “System Gold 126” (Beckman Coulter, Brea, USA), autosampler “Triathlon” (Spark), fluorescence detector “RF-10A XL” (Shimadzu, Kyoto, Japan) with SS420x interface set for excitation wavelength at 410 nm and for emission wavelength at 654 nm, online degasser (ERC3415 alpha, ERC), column thermostat Jet-Stream Plus set at 30 °C (Thermotechnic Products), column LiChroCART250-4 with Purospher STAR RP-18 endcapped and guard column LiChroCART4-4 with Purospher STAR RP-18e endcapped (Merck). The mobile phase was composed of acetonitril: 0.1 % trifluoroacetic acid in water (57.5:42.5 v/v) and the flow rate set at 1 mL/min. The retention time for mTHPC was about 10 min and the injection volume was 50 µL. The measuring range was from 0.25 to100 pg/µL (r^2^ = 0.9998) and the detection limit 0.05 pg/µL. The software used was 32 Karat Software, Version 5.0, Build 1021 (Beckman Coulter). The tissue concentration of mTHPC was determined from a calibration curve constructed by plotting the peak height of mTHPC standard solutions versus their concentrations. The calibration was linear within this range.

### In vivo PDT

Before treatment 90 tumor-bearing mice were injected subcutaneously with 1.5 mg/kg bw of the painkiller Metacam (Kantonsapotheke). Subsequently they were intravenously injected with one of the drug formulations (≙0.15 mg mTHPC/kg bw) and treated at the optimum time point according to the biodistribution study. For laser irradiation the mice were covered with a surgical drape, leaving only the tumor unprotected (≙ an irradiation area of 1.5 cm in diameter). Mice were held restrained tightly and irradiated with a Ceralas PDT laser 652 (Biolitec) for either 100 or 200 s (≙10 or 20 J/cm^2^; 100 mW/cm^2^). To monitor treatment effects tumor sizes were measured with a Vernier Caliper every 3 days and all mice were photographed with an 8 MP camera (Samsung, Seoul, South Korea) before treatment and up to 14 days later.

### Histology and Immunohistochemistry

In order to screen for short term and long term PDT effects 48 h and 14 days after laser irradiation half of the mice (n = 45) were sacrificed in each group. Liver, kidney, spleen and tumor were taken and rinsed with PBS. The organs were subsequently fixed with four per cent formaldehyde (FA)/PBS for 12 h and transferred to PBS or snap frozen in liquid nitrogen. FA fixed samples were dehydrated with an increasing alcohol series and embedded in paraffin. Five micrometer sections were cut and transferred to Superfrost glass slides (Thermo Fisher, Waltham, USA). The sections were deparaffinized and either stained with haematoxylin and eosin or processed for immunohistochemistry. For the latter the slides were washed repeatedly in Tris buffered saline (TBS) and blocked for 30 min in 1 % bovine serum albumin (BSA)/TBS. The slides were incubated with an anti-ki-67 antibody (Abcam, #ab15580, Cambridge, UK) over night at four degrees Celsius (1:100 in TBS). All following steps were performed at room temperature. After another washing step with TBS anti-rabbit-biotin antibody (BioScience Products AG, Emmenbrücke, Switzerland) was added (1:100 in BSA/TBS) for 30 min. After washing with TBS, the slides were incubated with Streptavidin Peroxidase (Biospa, Milano, Itlay, 1:100 in TBS) for 30 min. Another washing step with TBS followed, then the endogenous peroxidase was blocked by placing the slides in 0.3 % H_2_O_2_/TBS for 20 min. After another washing step with TBS the slides were incubated with 0.7 mg/mL 3,3′-diaminobenzidine/H_2_O for 3–20 min. The slides were washed with dH_2_O and mounted with glycergel (Dako, Glostrup, Denmark).

### Quantitative reverse transcriptase polymerase chain reaction (qRT-PCR)

Twenty mg frozen tumor tissue was transferred to MagNALyser Green Beads tubes (Roche, Basel, Switzerland) and 600 microliter lysis buffer (RNeasy Mini Kit, Qiagen, Venlo, The Netherlands) was added. Tissue homogenization was carried out according to manufacturer’s instructions with a Precellys 24 Homogenizer (Bertin, Montigny le Bretonneux, France). The lysate was centrifuged for 1 min at 11.000*g* and transferred to RNeasy Mini Spin columns (Qiagen). RNA extraction was performed according to manufacturer’s protocols. Five hundred ng of purified RNA were subsequently used for cDNA synthesis with QuantiTect Reverse Transcription Kit (Qiagen), which was carried out according to the manufacturer’s recommendations. QRT-PCR was performed with hydrolysis probes from a Universal Probe Library (Roche) on a LightCycler 480 (Roche). The PCR program consisted of an activation phase of 10 min at 95 °C followed by 45 cycles with 15 s at 95 °C and 1 min at 60 °C. Data was analyzed with the LightCycler480 software and REST software (http://www.gene-quantification.de). Primer sequences are listed in Table 1.

**Table 1:** Sequences of primers

Gene	Primer forward 5′–3′	Primer reverse 5′–3′	Probe
Human GAPDH	CAGCAAGAGCACAAGAGGAA	GTGGTGGGGGACTGAGTGT	#3
Human TACSTD	AGAGAGGGAGTGAGAGAAATTAAGG	GCGACTCCCTTTTCGTTCTT	#23
Human MMP7	GCTGACATCATGATTGGCTTT	TCTCCTCCGAGACCTGTCC	#72
Human ALDH1A3	TGGTGGCTTTAAAATGTCAGG	TATTCGGCCAAAGCGTATTC	#53
Human MKI67	CCAAAAGAAAGTCTCTGGTAATGC	CCTGATGGTTGAGGCTGTTC	#39
Human GLUT1	CTTTTCGTTAACCGCTTTGG	CGAGAAGCCCATGAGCAC	#62

Probe numbers relate to the Universal Probe Library (Roche)

*GAPDH* Glyceraldehyde 3-phosphate dehydrogenase; *TACSTD2* Tumor-Associated Calcium Signal Transducer 2; *MMP7* Matrix Metalloproteinase-7; *ALDH1A3* Aldehyde Dehydrogenase 1 Family, Member A3; *MKI67* Marker of Proliferation Ki-67; *GLUT1* Glucose Transporter 1

### Data analysis and statistics

For the measurement of tumor volumes (V) the following formula was used: $V = \frac{\pi }{6} \times L \times W^{2}$; where L corresponds to the length of the tumor and W to width of the tumor.

All groups consisted of at least five individuals.

Measurement raw data was transformed by square root transformation and 1-way ANOVA was performed on data sets of day zero, five and 14 after treatment.

## Results

The HNSCC model in nude mice worked best with a subcutaneously injected inoculation volume of 100 μL ringer lactate solution containing 1.5 × 10^6^ CAL-33 cells. While for the concentrations 1 × 10^6^ and 2 × 10^6^ cells no exponential tumor growth phase was reached (Additional file 1: Fig. S1 A,C), after injection of 1.5 × 10^6^ cells solid tumors with a calculated average volume of 150 mm^3^ developed within 24 days (Additional file 1: Fig. S1 B).

**Fig. 1:**
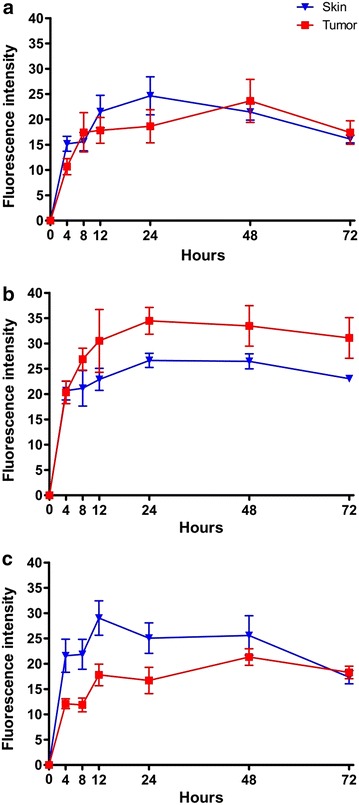
Spectrometric fluorescence measurements in skin and tumor after injection of Foscan (**a**), Foslip (**b**) and Lipidot (**c**)

The biodistribution profile established by spectrometric fluorescence measurements of mTHPC revealed that all formulations accumulated in the tumor but the distribution patterns were different for the three substances (Fig. 1). After Foscan injection the fluorescence in the tumor increased fast until 8 and then the curve reached a plateau. Tumor and skin accumulations were rather similar in trend but after 8 h, 48 h and 72 h slightly higher tumor fluorescence could be detected. Therefore 8 h was selected as the optimum drug–light interval for Foscan (Fig. 1a). Foslip accumulation in the tumor rose sharply until 12 h with the curve flattening afterwards. The detected fluorescence was higher in the tumor when compared to skin between 24 and 72 h. Accordingly 24 h was chosen as ideal treatment timepoint for Foslip (Fig. 1b). Lipidots accumulated strongly in the skin, peaking at 12 h after injection. Fluorescence accumulation within the tumor was increasing over time but was delayed when compared to skin. Fourty eight hours after injection Lipidots started to be cleared from the skin while accumulation within the tumor persisted. Although accumulation in the tumor was not higher 72 h was chosen as drug–light interval when less Lipidots were present in the skin (Fig. 1c). As a result of these fluorescence biodistribution profiles, the drug–light interval selected and applied for all further in vivo experiments are 8 h for Foscan, 24 h for Foslip and 72 h for Lipidot.

**Fig. 2:**
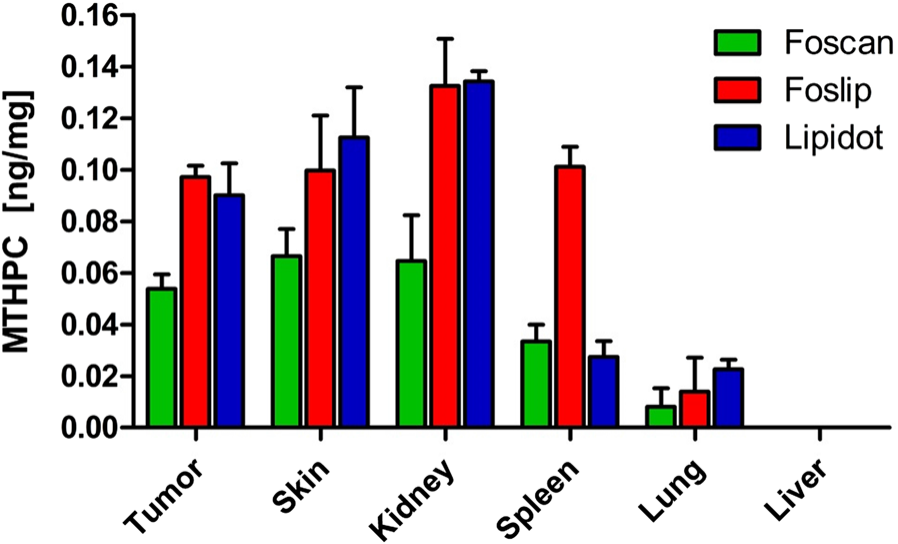
Tissue concentration of mTHPC (ng/mg wet tissue weight) 72 h after injection, as determined by HPLC analysis

HPLC analysis confirmed mTHPC accumulation in the tumor 72 h after injection of all drug formulations (Fig. 2). Concentrations of Lipidots and Foslip were comparable, while the mTHPC concentration was lower with the Foscan formulation at this time point. Kidneys as well as skin showed high accumulation with Lipidots and Foslip and lower accumulation with Foscan. Foslip concentration was also high in the spleen whereas Lipidots and Foscan were present in this organ to a much lower extent. Very low concentrations were found in the lung with all three formulations and no drug could be detected in the liver with either formulation at 72 h.

**Fig. 3:**
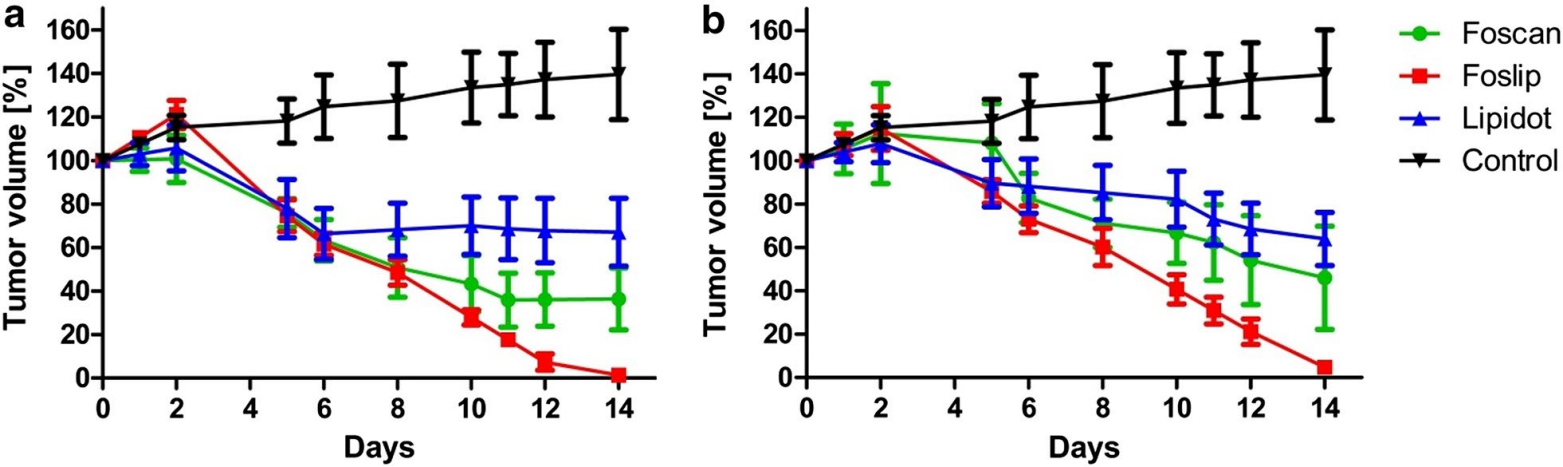
Caliper measurements of tumor volume changes after PDT (day 0) with 10 J/cm^2^ (**a**) and 20 J/cm^2^ (**b**)

Therapeutic effects after PDT treatment, analyzed by tumor size measurements indicated a treatment response to all three drug formulations (Fig. 3). The best results were accomplished by Foslip induced PDT which finally resulted in complete tumor remission with both light doses (10 and 20 J/cm^2^; 100 mW/cm^2^) (Fig. 3a, b). Foscan-PDT was also effective but tumors stopped to decrease further in size after 12 days with the lower light dose of 10 J/cm^2^ (Fig. 3a). Tumor residues of around 40 % of the initial tumor volume (i.e. before treatment) were still present 14 days later with both light doses (Fig. 3a, b). Lipidots, although diminishing the tumor masses significantly failed to decrease the tumor size further after 6 days with the lower light dose (Fig. 3a). The higher light dose (20 J/cm^2^) resulted in continuous reduction of tumor masses down to around 60 % of the initial tumor volume (Fig. 3b). Fourteen days after treatment Foslip was significantly superior to both, Foscan and Lipidots at lower light doses (p < 0.05) and significantly superior to Lipidots at higher light doses (p < 0.01).

**Fig. 4:**
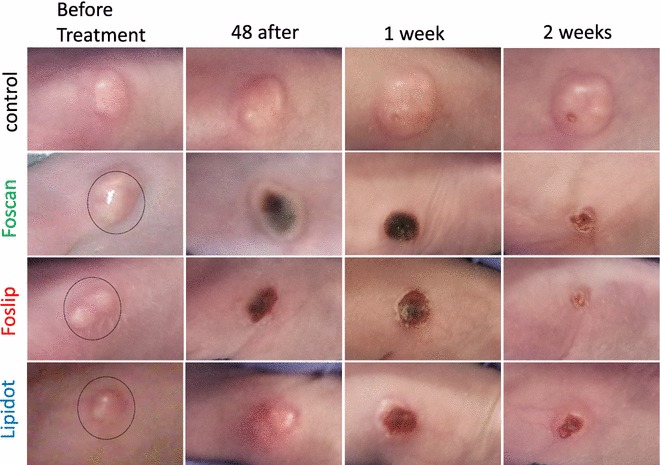
Images of tumors after PDT with 10 J/cm^2^. The irradiation area had a diameter of 1.5 cm (*circle*)

**Fig. 5:**
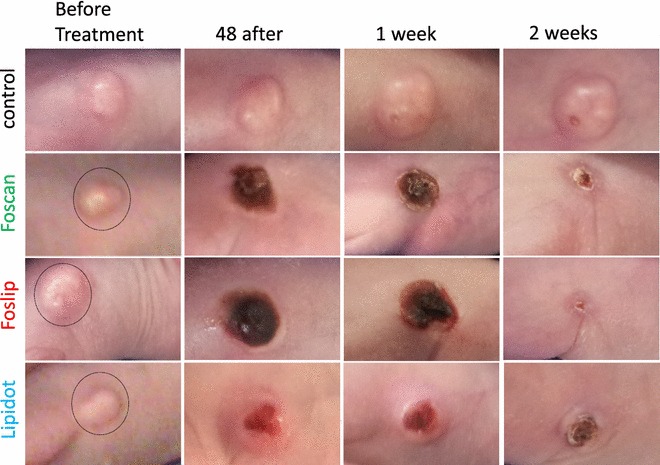
Images of tumors after PDT with 20 J/cm^2^. The irradiation area had a diameter of 1.5 cm (*circle*)

Fourty eight hours after Foscan and Foslip mediated PDT skin burns were visible at the irradiated spot with both light doses but only slight burns occurred after Lipidot-PDT, even with the higher light dose (Figs. 4, 5). One week after PDT necrotic tissue and crusts were visible with all three drug formulations at both light doses. Fourteen days after Foslip-PDT visible tumor masses had disappeared completely and the skin had healed with minimal scarring. Tumors treated with Foscan-PDT had diminished significantly in size and the skin had started to heal but small crusts and residual tumor tissue remained. Lipidot-PDT treated tumors had also diminished in size after 14 days but with this formulation an outer rim of the tumor remained with a crust from destroyed tissue in the middle. Generally the destructive effects as well as the burning of the skin were more severe with the higher light dose in all cases which, however, did not seem to affect healing negatively.

**Fig. 6:**
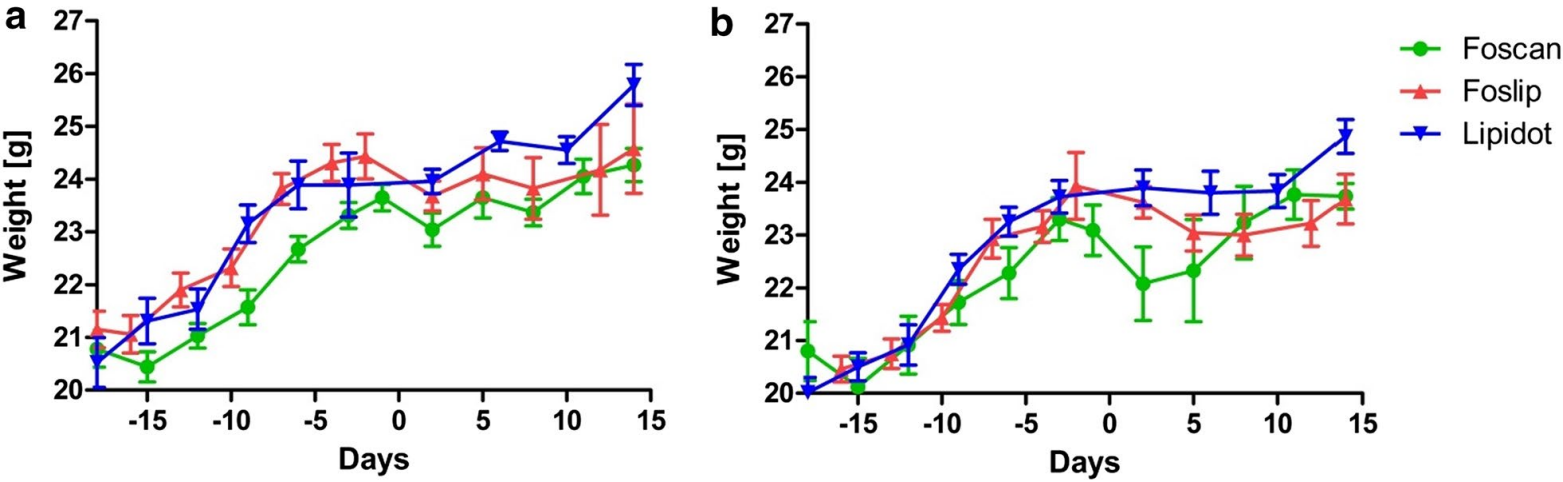
Body weight changes before and after PDT (at day 0) with 10 J/cm^2^ (**a**) and 20 J/cm^2^ (**b**)

A drop of body weight was observed in mice after Foscan and Foslip mediated PDT but more severe in case of Foscan (Fig. 6a). Also the higher light dose lead to a stronger body weight drop (Fig. 6b). Lipidots on the other hand did not result in any loss of body weight with neither light dose. If anything it delayed the body weight gain of the juvenile mice slightly (Fig. 6a, b).

A change in behavior of mice during and after administration of Foscan was apparent. The mice curled upon injection, which seemed to be painful to the rodents. Also during and after PDT the mice struggled and tried to avoid strongly to be touched, despite being treated with analgesics. Foslip and Lipidot injections as well as PDT seemed to be well tolerated with mice not showing any unusual behavior.

**Fig. 7:**
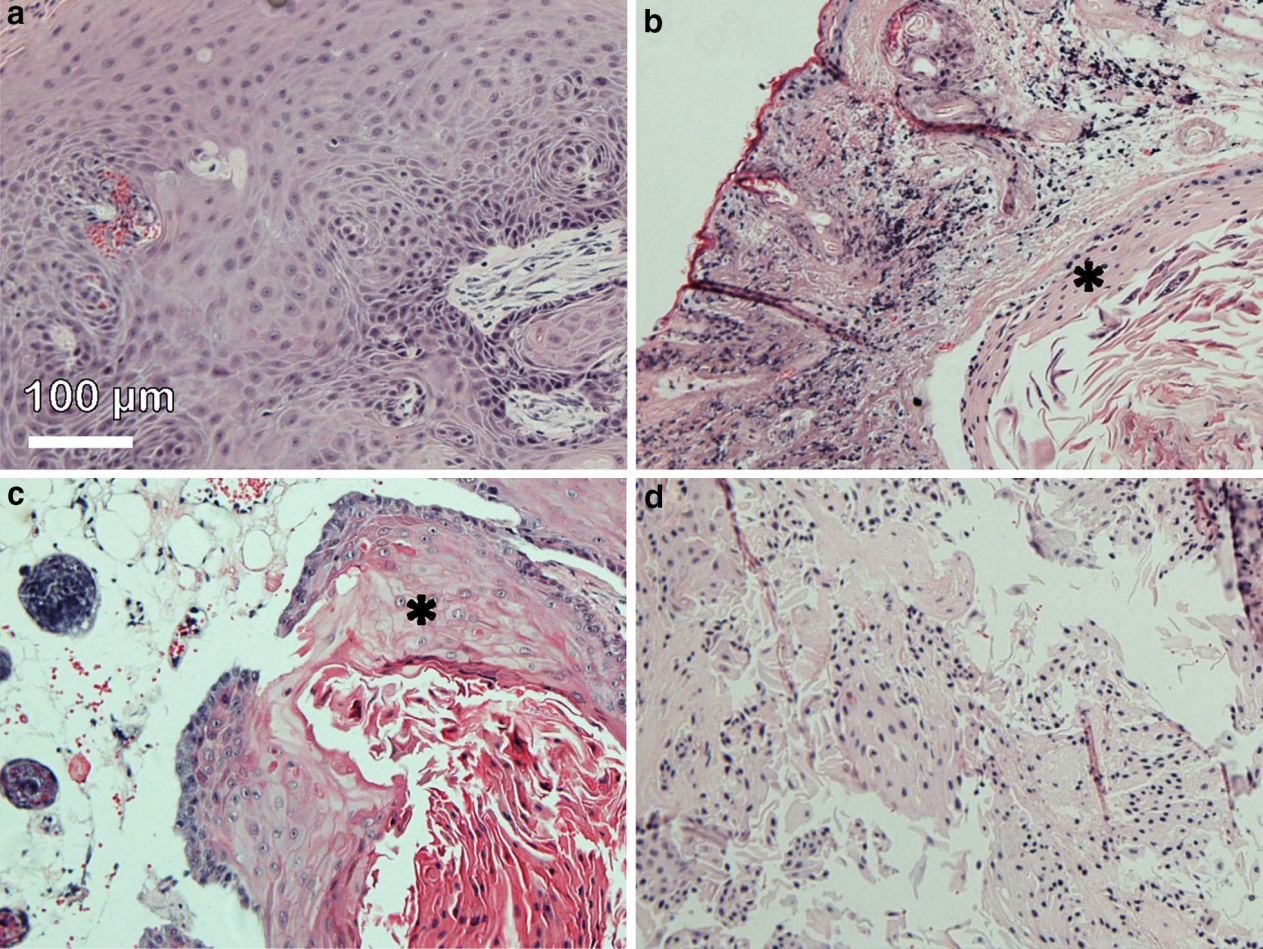
H&E stain of CAL-33 tumors. **a** Untreated control tumor. Tumor 48 h after PDT with Foscan (**b**), Lipidot (**c**) and Foslip (**d**). Laser light irradiation 20 J/cm^2^. *Asterisk* (**b**, **c**) : tumor tissue. **a**, **d**: only tumor tissue

Histological analysis was in accordance with caliper measurements revealing vascularized vital CAL-33 tumors in untreated mice (Fig. 7a). Fourty eight hours after Foscan mediated PDT the tumors showed clear features of destruction with lamellar appearing tumor parts and flattened cells (Fig. 7a). Lipidot-PDT created the same lamellar features but a larger area in the outer part of the tumors appeared to be left intact (Fig. 7c). Foslip-PDT, however, led to lamellar parts and strongly flattened cells throughout the whole tumor mass (Fig. 7d).

**Fig. 8:**
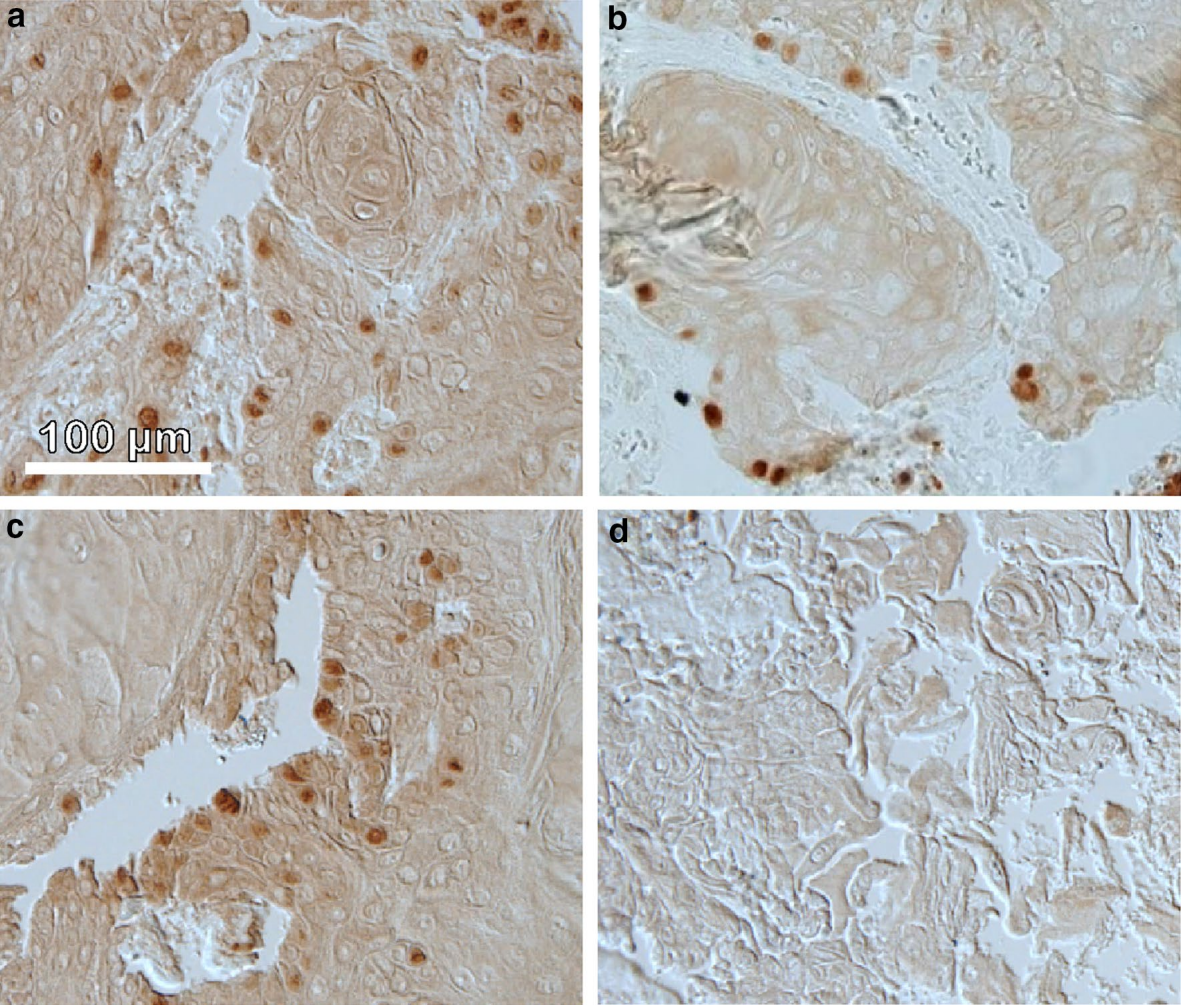
Ki-67 immunohistochemistry for CAL-33 tumors. **a** Untreated control tumor. Tumor 48 h after PDT with Foscan (**b**), Lipidot (**c**) and Foslip (**d**). Laser light irradiation 20 J/cm^2^

To distinguish between vital, proliferating cancerous and damaged tumor tissue a proliferation marker (ki-67) was used (Fig. 8). Antibody staining supported the tumor size measurements confirming diminished proliferation corresponding to less ki-67 expressing cells 48 h after PDT with all formulations. Foscan-PDT treated tumors showed little ki-67 positive cells when compared to untreated tumors after 48 h (Fig. 8b). Tumors after Lipidot mediated PDT still exhibited several ki-67 positive cells but less than untreated controls (Fig. 8c) and tumors that had been subjected to Foslip-PDT exhibited no ki-67 stained cells at all (Fig. 8d).

Histological and immunohistochemical analyses of tumors 14 days after treatment supported caliper measurement data. Tumors which had been subjected to Foslip-PDT were completely eradicated just leaving fibrotic scar tissue behind. Tumors after Foscan-PDT were not fully destroyed leaving some tumor tissue intact while the tumors after Lipidot-PDT were only partly destroyed with some cells positive for ki-67, thus still proliferating (data not shown).

Possible side effects of the treatments were investigated by analyzing livers, kidneys and spleens 48 h and 14 days after PDT. Liver damage was recognizable by deformed blood vessels and condensed nuclei of hepatocytes 48 h after Foscan and Foslip mediated PDT but not after Lipidot-PDT. However, the morphological changes were reversible as 14 days later all livers displayed similar morphological appearance. No damage of other organs was detectable neither 48 h after nor 14 days after PDT.

**Fig. 9:**
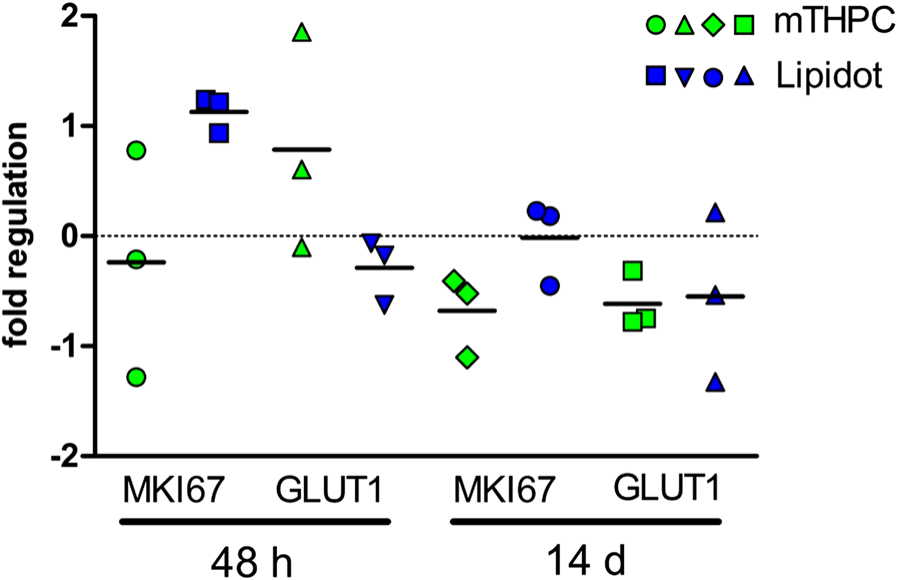
QRT-PCR data from tumors 48 h and 14 days after mTHPC-PDT and Lipidot-PDT. Laser light irradiation 20 J/cm^2^

We tested possible expression changes of five genes (TACSTD, MMP7, ALDH1A3, MKI67, GLUT1) in tumors 48 h and 14 days after mTHPC and Lipidot mediated PDT compared to untreated tumor controls (Fig. 9). Foslip-PDT destroyed the tumors completely and therefore no RT-PCR analysis was performed. MMP7 and ALDH1A3, that are stem cell markers for squamous cell carcinoma [29, 30], were not expressed in neither treated nor untreated tumors. TACSTD as marker for tumor aggressiveness [31] was not present in relevant abundance either. GLUT1, that may reflect the grade of malignancy [32], showed upregulation 48 h after mTHPC mediated PDT but not after Lipidot-PDT. However, these alterations were not present 14 days later. The proliferation marker gene MKI67 [33] did not reveal a significant expression change although it was expressed to a slightly higher extent in tumors after Lipidot-PDT when compared to tumors that had been exposed to Foscan-PDT.

## Discussion

Many preclinical studies provide evidence that PDT has great potential as anti-cancer modality. However, the hurdles of limited solubility of photosensitizers and photosensitivity of patients hamper routine use in the clinics and made encapsulation of PSs into nanocomposites an attractive option. Recently, the successful encapsulation of mTHPC in several nanocarriers had been described, such as polymeric nanoparticles [34, 35] and calcium phosphate nanoparticles [36]. In a similar approach in our former studies we presented the successful encapsulation of mTHPC into fully biocompatible and biodegradable lipid nanoemulsions and showed first data in monolayer cells [23, 24]. In our subsequent investigations in multicellular tumor spheroids we identified a formulation with a high mTHPC payload that featured the same excellent PDT effects as the free mTHPC but with a reduced dark toxicity (Lipidots) [25]. This nanoemulsion consists of a phospholipid (lecithin) monolayer, protected by a PEG-shell with a soybean/wax core, where mTHPC is incorporated. The average particle diameter of the most promising formulation was 50 nm with a PDI of 0.17 and a zeta potential close to −7 mV.

In the present study we now report for the first time on in vivo PDT with these novel PS-loaded Lipidots using a cancer xenograft nude mouse model. To better compare PDT effects of Lipidots, we included treatments with the conventional and approved mTHPC formulation Foscan [37–39] as well as the liposomal mTHPC formulation, Foslip, that already gave promising results in the treatment of cancer in cats [28, 40] and mice [40–42]. The lipsomes in the Foslip solution had an average particle size of 135 nm and a PDI of 0.089 with a zeta potential of around -13 mV.

One of the prerequisites for successful PDT is a high accumulation of the PS within the tumor site. While it is known that PSs are preferentially taken up by cancer cells compared to normal cells [13], in vivo intratumoral doses of PSs may actually often be low due to solubility problems and/or interactions with plasma proteins [42]. Increasing the PS dose, however, bears the risk of high circulating PS amounts and photosensitivity of skin and eyes.

We here showed that after intravenous injection Foscan accumulates in cancer xenografts and that treatment with Foscan-PDT significantly reduced tumor volumes under the selected conditions. However, our histological analyses confirmed that Foscan-mediated PDT did leave tumor residues behind in most cases. These residues were still present 14 days after treatment. Our HPLC data indicated a lower cancer accumulation of Foscan when compared to the other formulations 72 h after injection. The low accumulation, probably due to solubility problems, might be an explanation for the suboptimal PDT success. Also the other organs presented with less mTHPC content in the case of Foscan. Apart from solubility issues, an interaction with plasma proteins or, when taking into account the late time point of the HPLC measurement, faster systemic clearance could be at work.

Immunohistochemistry with ki-67 antibodies revealed that after Foscan-PDT, proliferating cells were still present in these samples but not more than in untreated cancers. Since elevated ki-67 is thought to be indicative for an unfavorable prognosis in head and neck cancers [43], incomplete PDT apparently did not select for this phenotype. To further characterize surviving tumor cells after PDT with Foscan, we performed qPCR studies for selected genes. Gene expression analyses revealed that cancer stem cell markers MMP7 and ALDH1A3 were neither transcribed before nor after PDT in CAL-33 cells. However, one genetic marker of tumor aggressiveness, GLUT1, was upregulated after Foscan-PDT. To the best of our knowledge, this is the first report on an increase of GLUT1 mRNA due to Foscan-PDT. The upregulation may have been the result of a PDT-related acute stress response as GLUT1 has previously been described as cell stress response gene [44]. However, 14 days after Foscan-PDT GLUT1 expression returned to control levels, suggesting that no permanent cell transformation into a GLUT1-related aggressive subtype had taken place. Although in our model remaining tumor cells did not start to proliferate in an aggressive manner we cannot exclude that they changed their phenotype. It is known that PDT can cause resistance in tumor cells under certain conditions and an acute stress response is one of them [11]. Thus, it would be interesting to investigate PDT resistance mechanisms in a follow up study by irradiating the tumors repeatedly.

Despite the different nature of the nanoformulations both, Lipidots and Foslip, accumulated in the xenografts and could reduce tumor volumes significantly after PDT. The observed slower cancer accumulation rate of Lipidots compared to Foscan is in line with our previous in vitro results from cancer spheroids [25] where penetration of Lipidots into the spheroid core was delayed. However, while tumor residues were still present after Lipidot-PDT, Foslip mediated PDT could eradicate the cancers completely. After Lipidot-PDT primarily the outer rim of the tumor seemed to be left intact, which was confirmed by histological analyses. Interestingly, we have observed a similar outcome already in our previous in vitro study with multicellular tumor spheroids [25] where spheroids were dying in the center but outer cell layers were left intact. The reason for this phenomenon is not clear yet. We can only speculate that e.g. the cells in the center are more susceptible to PDT due to poor nutrient supply or that in the outer layers a kind of quenching effect might occur. Another possible explanation could be that the particles stay intact when entering the cell and are therefore less accessible for light activation. Ki-67 staining also revealed proliferating cells after Lipidot PDT as seen in the case of Foscan, while after Foslip PDT no such cells were present. QRT-PCR data showed no GLUT1 upregulation after PDT for neither of the particles, indicating that encapsulation changes certain cellular effects of the PS in vivo. This result is well in line with our previous in vitro results where we could show that encapsulation into Lipidots can dampen the expression response after mTHPC mediated PDT for a wide range of genes [25]. With regard to drug resistance encapsulation might thereby offer an advantage over the use of the free formulation.

Both particles, Lipidots and Foslip, contain the same drug and drug amounts were kept constant for all formulations. Furthermore, 72 h after injection quantitative HPLC analysis showed similar mTHPC amounts in the xenograft for both nanoformulations. According to these observations the difference in therapy outcome is most likely due to the nanoparticle itself.

While in the Foscan solution mTHPC is solubilized by use of a solvent consisting of propylene glycol/alcohol in the liposomes the PS, because of its amphiphilic nature, is entrapped in the phospholipid bilayer. In contrast in the nanoemulsion (Lipidots) with a phospholipid monolayer hull the PS is incorporated into the oil/wax core. Due to the different structure cells might interact with each nanoparticle in a different way and might cause or might not cause PS release by an uptake event. Furthermore, it is well known that the nature of the particle can have a major impact on its later subcellular localization, which is an import factor for the success of PDT [45]. Additionally both particles consist of different lipids and contain different lipid amounts in their formulation which is fivefold higher in the case of Lipidots.

From our biodistribution studies we have chosen drug–light intervals of 8 h and 24 h for Foscan and Foslip, respectively. To avoid high PS levels in the skin, for Lipidots, we extended the drug–light interval to 72 h. The idea behind this approach was to minimize the damage to healthy tissue by irradiation and to reduce the risk of subsequent scarring. However, with careful shielding as in our study, an earlier treatment time point might have been advantageous, since it has been shown that the drug–light interval determines in which compartment the PS accumulates preferentially. It has been demonstrated e.g. by Lassalle et al. [46] that at early time points Foslip is circulating mostly within the blood stream, only after several hours it can reach the tumor site and at later time points it is mainly located within the cancers of mammary carcinoma bearing nude mice. It might have been the case that at the late treatment time point blood vessels were not affected by PDT, which would have been necessary to avoid subsequent tumor regrowth. In the present study, biodistribution was investigated by two complementary methods, i.e. spectrometric fluorescence in vivo measurements and HPLC endpoint analyses. Clearly, certain discrepancies between results of these methods arose that may have been caused by different optical measurement conditions after injection of the formulations that were most likely influenced by differences in subcellular distribution, aggregation behavior or localization of the PS within the tissue. It is well known that the nature of the nanocarrier may have a strong influence on these parameters, which can in turn lead to decreased detectability by methods that rely on fluorescence and might ultimately hamper a treatment response. Considering the different size and composition of the nanocarriers a distinctively different biodistribution profile was to be expected.

However, differences in the detectability made it very difficult to predict the optimal treatment time point. On the other hand, use of HPLC detection for all treatment time points would have called for a huge amount of rodents, which we deliberately refrained from. Nevertheless a better correlation between fluorescence measurements and HPLC should be established for future experiments.

Furthermore, it has to be stated that for successful PDT, dosimetry, that means the appropriate drug and light dose, as well as the optimal drug–light interval are of uttermost importance. In the case of Foscan and Foslip appropriate treatment schemes had already been established previously [28, 46], while for Lipidots no such protocol exists. We therefore cannot exclude that the drug–light interval used in our mouse model was not optimal for all substances, especially for the Lipidot formulation.

A factor which is often neglected in the therapy of cancer patients is their overall constitution. Because their body is already massively weakened by the disease it is of uttermost importance that the administered drugs do not unnecessarily deteriorate their health status by causing additional side effects. When it comes to treatment tolerance Lipidots were clearly performing best. Convenient, painless injection, no change in behavior of mice and no body weight loss indicate a highly biocompatible nanoformulation. In contrast Foscan was problematic in this regard, causing painful injections and severe weight loss. Foslip was also superior to Foscan in this matter. Injections were unproblematic; however a slight weight loss was apparent. Furthermore our HPLC data showed high spleen accumulation only for Foslip, which leads to the conclusion that it is particularly susceptible to elimination from the blood stream via the reticuloendothelial system. Given the fact, that the particles are not protected by a PEG layer, it is not surprising that they are inferior to Lipidots in this regard.

As evidenced by histology, both Foslip and Foscan caused acute liver toxicity while the Lipidots seemingly had no such effect. However, 14 days after treatment all livers morphologically recovered. This is in line with our HPLC data which showed that already after 72 h no mTHPC was detectable in the livers, pointing towards a fast liver clearance as it had already been shown by Rovers et al. [47].

The high skin accumulation of Lipidots after systemic injection was a surprising finding as not much can be found in the literature concerning this phenomenon. Moreover no real explanations are given. However, many studies about topical nanoparticle administration and skin distribution exist. One explanation for prolonged skin accumulation in a study by Mittal et al. is e.g. a possible accumulation in hair follicles [48]. It would be very interesting to study the distribution of Lipidots in the mouse skin to investigate if this is the case and to determine their exact localization. These results might further clarify the interestingly different particle behaviour of both nanoformulations.

We also detected high mTHPC accumulation in the kidney with both nanoformulations. Bearing in mind that both intact particles are generally regarded as being too big for fast effective renal clearance [49] it is not surprising to still find mTHPC traces in this organ after 72 h, as clearance may be retarded through encapsulation. Therefore it can be assumed that free mTHPC will be cleared faster from the kidneys. Furthermore the overall biodistribution of mTHPC is expected to be worse due to solubility issues and unspecific aggregation.

Interestingly, despite the high mTHPC load, the kidney did not show signs of histological impairment, neither did other organs. Although we did not test for specific markers of organ function, we propose that none of the mTHPC formulations used cause severe side effects in kidney, spleen, lung and liver.

## Conclusions

In conclusion we could confirm in our study the superiority of nanoformulations to the free PS mTHPC. Bearing in mind that the free substance is related to several issues such as poor bioavailability, solubility and increased photosensibility of patients the development of a potent nanoformulation is a necessity. We could show that Foslip on the one hand is very effective in destroying the tumors itself. However, because the Lipidots’ biocompatibility is outstanding and superior to the liposomes we declare further investigations and protocol optimization a priority in the future.

## Additional file

10.1186/s12951-016-0223-8 CAL-33 tumor model in CD1-*Foxn1* ^*nu*^ nude mice. Tumor growth after injection with 1 x 10^6^ CAL-33 cells (A), 1.5 x 10^6^ CAL-33 cells (B) and 2 x 10^6^ CAL-33 cells. **Table S1.** Physicochemical characterization data of Lipidots.

## Abbreviations

PDT: photodynamic therapy
PS: photosensitizer
^1^O_2_: singlet oxygen
MTHPC: m-tetrahydroxyphenylchlorin
HPLC: high performance liquid chromatography
QRT-PCR: quantitative reverse transcriptase polymerase chain reaction
ROS: reactive oxygen species
PDD: photodynamic diagnosis
EPR: enhanced permeability and retention
DPPC: 1,2-Dipalmitoyl-sn-glycero-3-phosphocholine
DPPG: 1,2-Dipalmitoyl-sn-glycero-3-phosphorylglycerol sodium salt
PDI: polydispersity index
PEG: polyethylene glycol
PBS: phosphate buffered saline
IVC: individually ventilated cage
SPF: specific pathogen free
BW: body weight
FA: formaldehyde
TBS: tris buffered saline
BSA: bovine serum albumine
CDNA: complementary DNA
ANOVA: analysis of variance
HNSCC: head and neck squamous cell carcinoma
GAPDH: Glyceraldehyde 3-phosphate dehydrogenase
TACSTD2: Tumor-Associated Calcium Signal Transducer 2
MMP7: Matrix Metalloproteinase-7
ALDH1A3: Aldehyde Dehydrogenase 1 Family, Member A3
MKI67: Marker of Proliferation Ki-67
GLUT1: Glucose Transporter 1
