# Supplementary material for: Lipid nanoemulsions and liposomes improve photodynamic treatment efficacy and tolerance in CAL-33 tumor bearing nude mice
Source: J Nanobiotechnology. 2016 Oct 3;14:71. doi: 10.1186/s12951-016-0223-8 (PMC5048629; doi:10.1186/s12951-016-0223-8)
Supplement: Supplementary file 1 — 10.1186/s12951-016-0223-8 CAL-33 tumor model in CD1-Foxn1 nu nude mice. Tumor growth after injection with 1 x 10^6^ CAL-33 cells (A), 1.5 x 10^6^ CAL-33 cells (B) and 2 x 10^6^ CAL-33 cells. Table S1. Physicochemical characterization data of Lipidots. [file 12951_2016_223_MOESM1_ESM.docx]

**Supplementary Material**

*Nanoparticle preparation*

MTHPC was incorporated with a content of 920 molecules/particle. As observed in our preliminary studies^24,25^, mTHPC was efficiently encapsulated into lipid nanoparticles without affecting neither the colloidal properties of the carrier nor photophysical properties of the loaded PS. Indeed, an aggregation of mTHPC inside the lipid core of nanoparticles can be observed only for 50 nm particle at high payload (> 4%w/w total lipid, data not published). Estimated from the whole excipients initially incorporated in the Lipidot formulation, mTHPC was loaded in our study at 2.8 and 2.2 % w/w for 50 nm Lipidots (Table 1).

*Particle size and size distribution of lipid nanoparticles*

Dynamic light scattering (DLS) technique was used to determine the particle hydrodynamic diameter (in nm), particle size distribution (expressed by polydispersity index PDI) using Zetasizer Nano ZS (Malvern Instruments, France). At least three different nanoparticle preparations (lipid dispersed phase weight fraction: 10%) are measured per condition. Data were expressed as mean ± standard deviation of 3 independent measurements performed at 25°C. Only batch 1 was used for in vivo experiments, although batch to batch differences are minimal (Table 1).

Table 1. Physicochemical characterization data of Lipidots.

|  | **[Lipid] (mg/mL)** | **Nbr of part/mL** | **mTHPC mol /particle** | **[mTHPC] (µg/mL)** | **Drug Loading** | **Hydrodynamic diameter (nm)** | **Polydispersity Index** |
| --- | --- | --- | --- | --- | --- | --- | --- |
| Lipidot Batch 1 | 50 | 7.27565 x 10^14^ | ~920 | 771 | 2.2 % | 50.0 ± 1.12 | 0.172 ± 0.02 |
| Lipidot Batch 2 | 50 | 7.27565 x 10^14^ | ~920 | 771 | 2.8% | 47.7 ± 1.10 | 0.153 ± 0.01 |


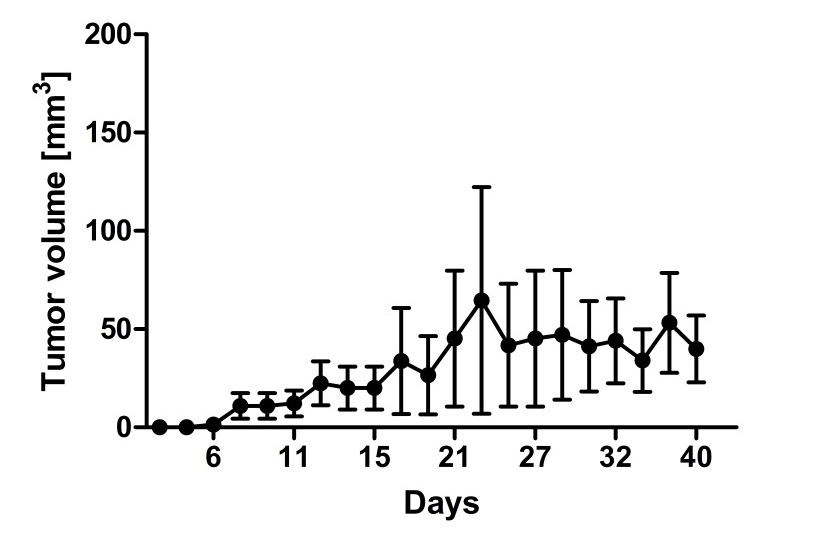


A


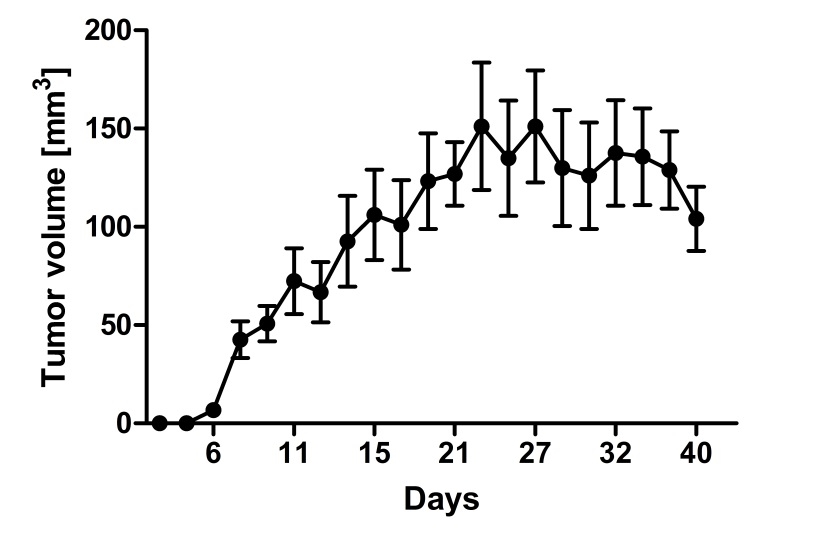


B


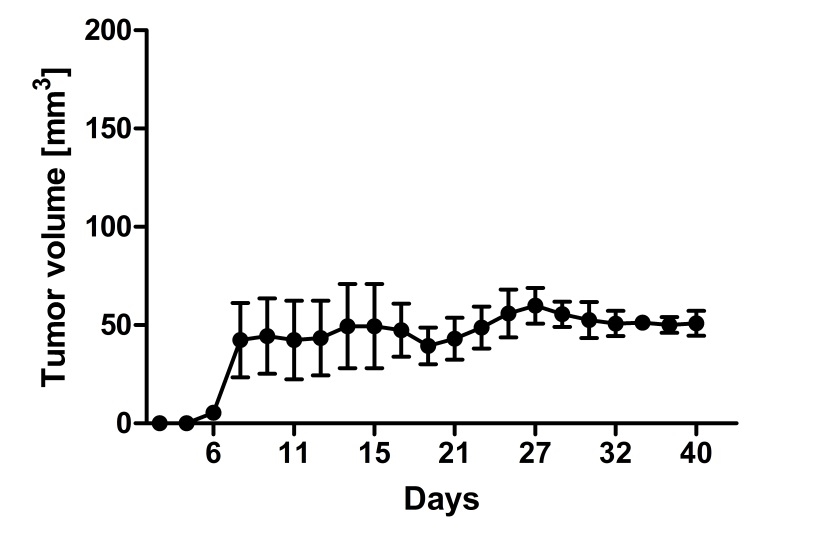


C

Fig.1. CAL-33 tumor model in CD1-*Foxn1^nu^* nude mice. Tumor growth after injection with 1 x 10^6^ CAL-33 cells (A), 1.5 x 10^6^ CAL-33 cells (B) and 2 x 10^6^ CAL-33 cells .
